# Liquid Chromatography–Single-Quadrupole Mass Spectrometry as a Responsive Tool for Determination of Biogenic Amines in Ready-to-Eat Baby Foods

**DOI:** 10.1007/s10337-018-3527-z

**Published:** 2018-05-02

**Authors:** Anna Czajkowska-Mysłek, Joanna Leszczyńska

**Affiliations:** 1 Mass Spectrometry Laboratory, Wroclaw Research Centre EIT+, 147 Stabłowicka, 54-066 Wroclaw, Poland; 2 Faculty of Biotechnology and Food Sciences, Lodz University of Technology, 4/10 Stefanowskiego, 90-924 Lodz, Poland

**Keywords:** Baby food, Biogenic amines, Dansyl chloride, HPLC–APCI–MS, Ion-source fragmentation

## Abstract

Baby food has never been the object of biogenic amine profiling. The aim of this study was to develop a highly sensitive method for analysis of biogenic amines in ready-to-eat baby foods. The principle of the developed method involves high-performance liquid chromatography coupled to single-quadrupole mass spectrometry (HPLC–APCI–MS) of dansyl derivatives, presented also in comparison with common diode array and fluorescence detection systems. The confirmation of correct identification of derivatives was performed by in-source fragmentation of the product ion at 170 *m*/*z*, performed only in one MS analyzer. The method was used to identify the amine profile and quantify the putrescine, cadaverine, histamine, tyramine, spermidine, and spermine content in 68 ready-to-eat baby foods. The limits of detection and quantification were in the range of 0.07–1.67 and 0.2–5.0 ng mL^− 1^. The method enabled quantification of amines at ng/g level in almost all analyzed samples, without any preconcentration step. Amine recoveries of 86.0–105.2% were obtained with RSD ≤ 9.7%. The developed method could be used for quantification of the most frequently occurring BAs in foods including vegetables, fish, meat, or fruit at previously undetectable concentration levels, making the method multimatrix applicable and highly-sensitive.

**Electronic supplementary material:**

The online version of this article (10.1007/s10337-018-3527-z) contains supplementary material, which is available to authorized users.

## Introduction

It has been acknowledged for a long time that formation of certain chemicals may pose a risk to human health. Such chemicals are biogenic amines (BAs)—nitrogenous compounds mainly formed by decarboxylation of the corresponding amino acids by spoilage and other microorganisms, with the exception of polyamines, which can be formed in vivo by the amination and transamination of aldehydes or ketones [1, 2]. BAs can be found in all food products, particularly in those with high protein content [3]. Since BAs are thermo-stable compounds, they are present even in the heat-treated foods [4].

Potential adverse reactions among infants and young children could appear after consumption of food containing toxic BAs, but the available data’s on digestive disorders in children are limited [5]. Complementary foods intended for infants and young children under the age of 3 years old including ready-to-eat products are not currently screened for BAs. As a result, no data on the profiles and concentrations of individual BAs in baby foods are available. Such challenging analysis requires a new methodology, with lower LODs and greater chromatographic separation to be applicable for routine analysis.

The most common technique used for analysis of BAs, due to its sensitivity and selectivity, is HPLC coupled to various detection systems. The methods involve pre-/post-column derivatization, and UV or FL detection [6], evaporative light-scattering detector (ELSD) [7], or more often are performed with MS detectors [8]. Separation of BAs is generally performed on columns with alkyl chain also conducted in UPLC systems [9, 10], or on HILIC (Hydrophilic Interaction Liquid Chromatography) columns [11].

Each method consists of two basic steps: extraction from food matrices, with optional clean-up with SPE [12, 13] or µSPE [14], matrix solid-phase dispersion (MSPD) [11, 15], dispersive liquid–liquid microextraction (DLLME) [16, 17] and derivatization to appropriate compounds for the detection technique used. The derivatization step may be performed using many reagents, such as *o*-phthaldialdehyde (OPA), dansyl chloride (Dns-Cl), benzoyl chloride, dabsyl chloride, 4-chloro-3,5-dinitrobenzotrifluoride, 1,2-naphthoquinone-4sulfonate, 6-aminoquinolyl-*N*-hydroxysuccinimidyl carbamate, or *N*-hydroxy-succinimide ester [8]. Co-extractives like free amino acids might compete with BAs in the analytical process what might result in poor recovery rates. Therefore, it is necessary to use different extraction solvents (perchloric acid, trichloroacetic acid, hydrochloric acid) and perform LLE to remove the amino acids from the sample matrix.

BAs profiling is certainly a challenge, primarily due to the complexity of food matrices, the presence of free amino acids and compounds that could interfere with the analytes, the low concentration of BAs and in some cases significant differences in the concentrations of individual amines in the amine profile. Limits of detection reported for the real samples for the majority of the available analytical methods are generally in µg g^− 1^ (µg mL^− 1^) range with a few exceptions [18]. LC–MS is the most precise and sensitive method, but is still not widely employed, particularly for routine analysis of food [8]. Using tandem MS/MS detectors, the derivatization step could be excluded, but analysis needs to be performed in HILIC mode.

The aim of this work was to develop an HPLC–APCI–MS method to evaluate, for the first time, the BAs content in commercial ready-to-eat baby foods. A methodological requirement was to achieve much lower (1–2 orders of magnitude) LOQs in comparison with current methods for quantification of the most frequently occurring BAs in foods (vegetables, fish, meat or fruit), without additional sample preconcentration, making the method also multimatrix applicable. The HPLC–APCI–MS method could be used as a responsive analytical tool for the identification of possible food constituents, which might have the most allergy-like potential and elicit food adverse reactions among infants and young children.

## Materials and Methods

### Chemicals

LC–MS grade Acetonitrile (VWR, Radnor, PA, USA) was used, and water purified using a MilliQ Direct 8 system (Merck, Darmstadt, Germany). Glacial acetic acid, perchloric acid 60%, l-proline for biochemistry, trichloroacetic acid, diethyl ether, sodium carbonate anhydrous, and acetone for LC were acquired from Merck (Darmstadt, Germany). Ammonium formate, formic acid, dansyl chloride, and 1,7-diaminoheptane were purchased from Sigma-Aldrich (St. Louis, MO, USA). All certified materials, including histamine dihydrochloride, tyramine hydrochloride, cadaverine dihydrochloride, putrescine dihydrochloride, spermidine trihydrochloride, and spermine tetrahydrochloride, were purchased from Dr. Ehrenstorfer GmbH (Augsburg, Germany). Other chemicals, including sodium hydroxide micropills, were obtained from POCH (Gliwice, Poland). Ammonium acetate for HPLC was supplied by J.T. Baker (Deventer, The Netherlands).

### Samples

Sixty-eight samples of commercial ready-to-eat baby food preserves intended for infants (4–12 months) and young children (1–3 years old), produced by ten (coded from A to J) leading manufacturers available in Poland were purchased from local shops. The products, available as dinners, soups, or desserts were pasteurized by manufacturers and packed in glass jars (125–250 g) or plastic boxes/pouches (50–250 g). The samples contained 8–12% of fish (vegetable-based with fish, sample Nos. 1–23, 23 products), 10% of meat (vegetable-based with meat, sample Nos. 24–38, 15 products), vegetables (sample Nos. 39–53, 15 products), and fruit (sample Nos. 54–68, 15 products).

### Standards/Samples Preparation Procedure

Standard solutions of selected amines and ISTD (1,7-diaminoheptane) were diluted with 0.4 M HClO_4_. Stock solutions were stored at 4 °C for 3 months and calibration solutions prepared daily before analysis. The concentration of ISTD was maintained at 50 ng mL^− 1^. The calibration curves range was based on the concentration levels of individual BAs determined in the analyzed samples. Dansyl chloride solution was prepared in acetone just prior to use.

**Fig. 1:**
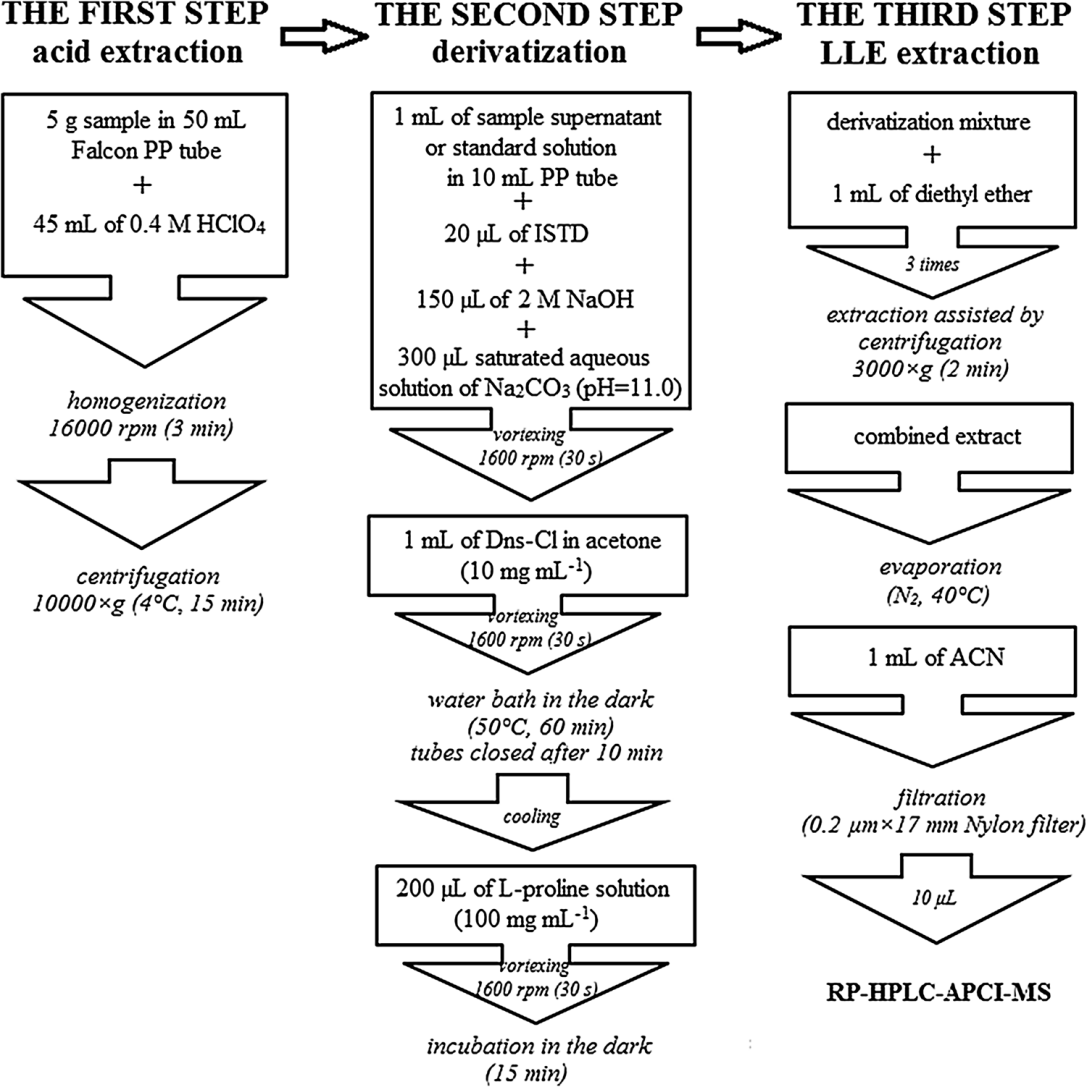
Sample preparation procedure flowchart

In the case of baby foods, two combined jars/boxes with diverse date codes were homogenized (if not homogeneous) using a laboratory mixer. The sample preparation procedure consists of three steps: an acid extraction, derivatization, and LLE extraction (Fig. 1).

The sample acid extracts could be stored at − 18 °C for approximately 6 months prior to further analysis. All samples were analyzed in triplicate.

### RP–HPLC–APCI–MS Conditions

Analyses were performed using HPLC–APCI–MS with selected ion monitoring (SIM) in positive ion mode. The HPLC system consisted of a Shimadzu Prominence UFLC liquid chromatography binary system equipped with two LC-20AD pumps, a DGU-20A3 degasser unit, an SIL-20ACHT autosampler, and a CTO-10ASVP thermostated column oven coupled to an LCMS-2020 detector, with an APCI interface, all supervised via CMB-20A controller.

Data analysis was performed using the LabSolution software (ver. 5.72 Shimadzu, Kyoto, Japan). MS acquisition was performed under the following conditions: APCI temperature 450 °C, nebulizing gas (N_2_) flow rate 4 L min^− 1^, drying gas (N_2_) flow rate 10 L min^− 1^, heat block temperature 300 °C, and desolvation line temperature 200 °C. The BAs were separated on a Gemini-NX C_18_ column (150 × 4.6 mm, 3 µm particle size, Phenomenex, Torrence, CA, USA), with a pre-column (4 × 3 mm) containing the same stationary phase, operated at 25 °C with a flow rate of 0.8 mL min^− 1^. The mobile phase consisted of 10 mM ammonium formate (A) and acetonitrile (B) with the following gradient elution program: 0.01–16.00 min 60–90% B; 16.01–24.00 90% B; and 24.01–30.00 60% B (re-equilibration).

HPLC analyses using different detection systems: an SPD-M20A Shimadzu diode array detector (connected in-line before MS) and a Shimadzu fluorescence detector RF-20A (offline MS) were also compared to establish the response of the detectors to the obtained dansyl derivatives. The conditions of HPLC–FLD analysis were as follows: mobile phase A (water)/ B (ACN), gradient 0–19.0 min 60–90% B, 19.0–20.0 min 90% B, 20.01 min 60% B, re-equilibration 27.0 min, flow rate 0.8 mL min^− 1^, column temperature 25 °C, 20 µL injection, *λ*_ex_/*λ*_em_ 352/515 nm.

### Method Validation and QA/QC Procedure

After selecting the optimum conditions for the sample preparation and HPLC–APCI–MS separation, method validation was performed. The method was validated on the most complex baby food matrix—vegetable with fish. The quantification of BAs was based on internal standard calibration. The regression equations were calculated as six-point calibration curves with weighting factor *1*/*x*, based on quantification of the ratio of the amine peak area to the peak area of the ISTD vs. the concentration of BA. The calculation of LODs and LOQs was estimated for the LC–MS technique and the method. LOQs were measured as the lowest concentrations at which the analyte could be detected reliably, and defined as concentrations resulting in a relative standard deviation (RSD) below 20%. They were thus a measure of the assay’s precision at low analyte levels. The LOQ values obtained were set as the lowest concentration levels on calibration curves. LODs were calculated from LOQ as LOD = LOQ/3.

The method was validated with regard to linearity, matrix effect, precision, and accuracy. To evaluate the linearity of the method, the standard solutions were prepared by diluting a specific volume of the stock standard to achieve several concentrations. To estimate the matrix effect, five concentrations of the standard were added (in triplicate) to a baby food containing endogenous BAs. The slopes of the calibration curves were compared with those obtained for standard solutions. The matrix effect was calculated as ratios of the slope of matrix-matched calibration curves (5-points) and the slope of the calibration curves in solvent (6-points) multiplied by 100 [19]. The quality assurance/quality control (QA/QC) samples were inserted into each batch (blank solvent, duplicates of the sample from the beginning of the batch at the end of batch). In addition, the stock solutions were used as the QC samples. The QC samples were prepared daily at the low, medium, and high concentrations of standard BAs solution in fish-based baby food extract, and were measured at the beginning or end of each batch. The RSD for the peak area was determined as a measure of precision. The precision of the method was assessed based on intra-day repeatability (one day, *n* = 3) and inter-day reproducibility (three consecutive days, *n* = 9), in triplicate analyses of sample spiked with approx. 25, 125, and 250 ng mL^− 1^ of PUT, SPD, and SPM and approx. 5, 25, and 50 ng mL^− 1^ of CAD, HIS, and TYR, respectively. The accuracy of the method was evaluated by quantifying the recovery of standard solutions. The recovery test was performed on laboratory made vegetable baby food with fish using the method of standard addition. The sample was spiked with high, intermediate, and low levels of standard BAs solution.

## Results and Discussion

### BA Profile of Ready-to-Eat Baby Foods

**Fig. 2:**
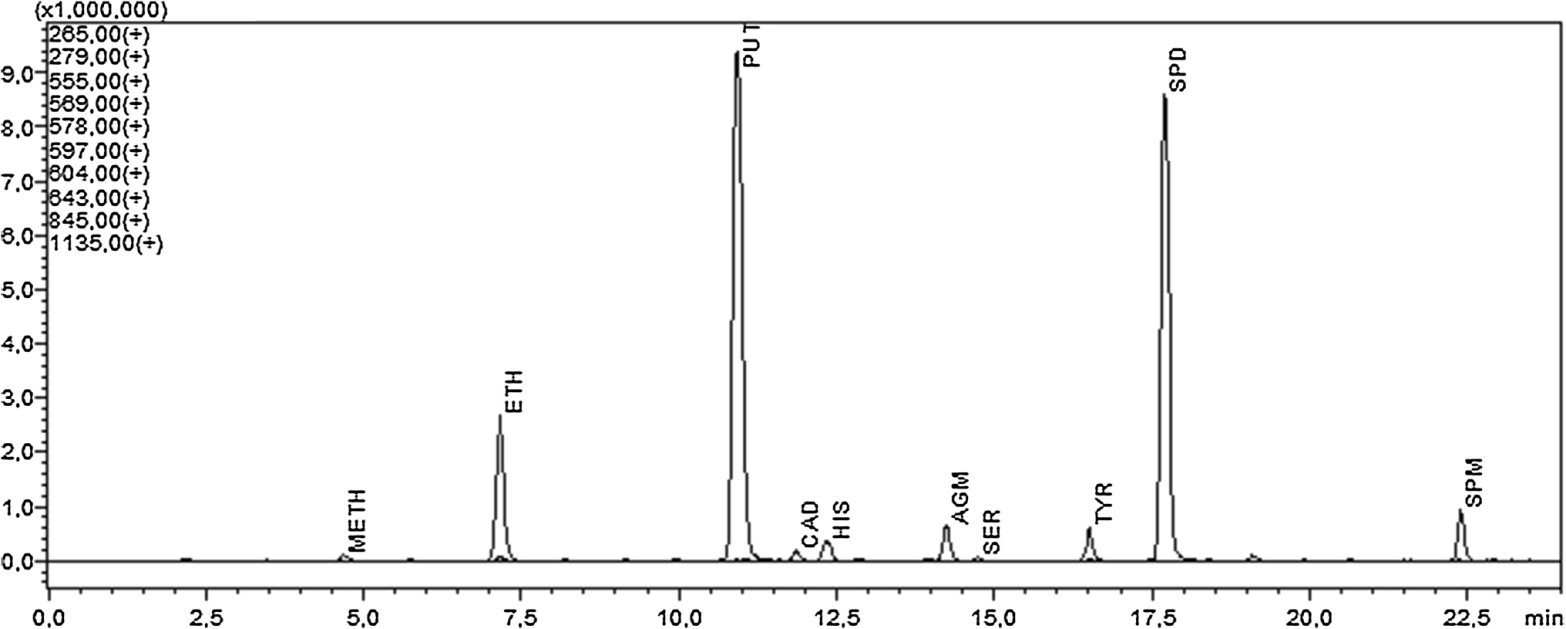
HPLC–APCI–MS (SIM+) chromatogram obtained in scan mode (*m*/*z* 100–1300) presenting the BA profile of baby food sample (No. 3). Peak identification: METH (methylamine), ETH (ethylamine), PUT (putrescine), CAD (cadaverine), HIS (histamine), AGM (agmatine), SER (serotonin), TYR (tyramine), SPD (spermidine), SPM (spermine)

The BA profile of commercial ready-to-eat baby foods consists mainly of ten amines (Fig. 2). The identified amines (HPLC–APCI–MS in full scan mode) were methylamine (METH, [M+H]^+^ = 265), ethylamine (ETH, [M+H]^+^ = 279), putrescine (PUT, [M+H]^+^ = 555), cadaverine (CAD, [M+H]^+^ = 569), histamine (HIS, [M+H]^+^ = 578), agmatine (AGM, [M+H]^+^ = 597), tyramine (TYR, [M+H]^+^ = 604), serotonin (SER, [M+H]^+^ = 643), spermidine (SPD, [M+H]^+^ = 845), and spermine (SPM, [M+H]^+^ = 1135). In analyzed samples, other psychoactive amines commonly found in food products including phenylethylamine (PEA, [M+H]^+^ = 355), tryptamine (TRP, [M+H]^+^ = 394) were not detected. Apart from the above-mentioned BAs, other psychoactive amines including dopamine (DOP, [M+H]^+^ = 853) and norepinephrine (NE, [M+H]^+^ = 869) have been detected, but only in fruit-based products. To evaluate the toxicity potential of ready-to-baby foods, only six amines were selected from BAs identified in their profile. These were selected due to their direct toxicity (HIS, TYR), their potential to enhance HIS toxicity (PUT, CAD) or their carcinogenic properties (SPD, SPM) [2].

### RP–HPLC–APCI–MS Method Development

**Fig. 3:**
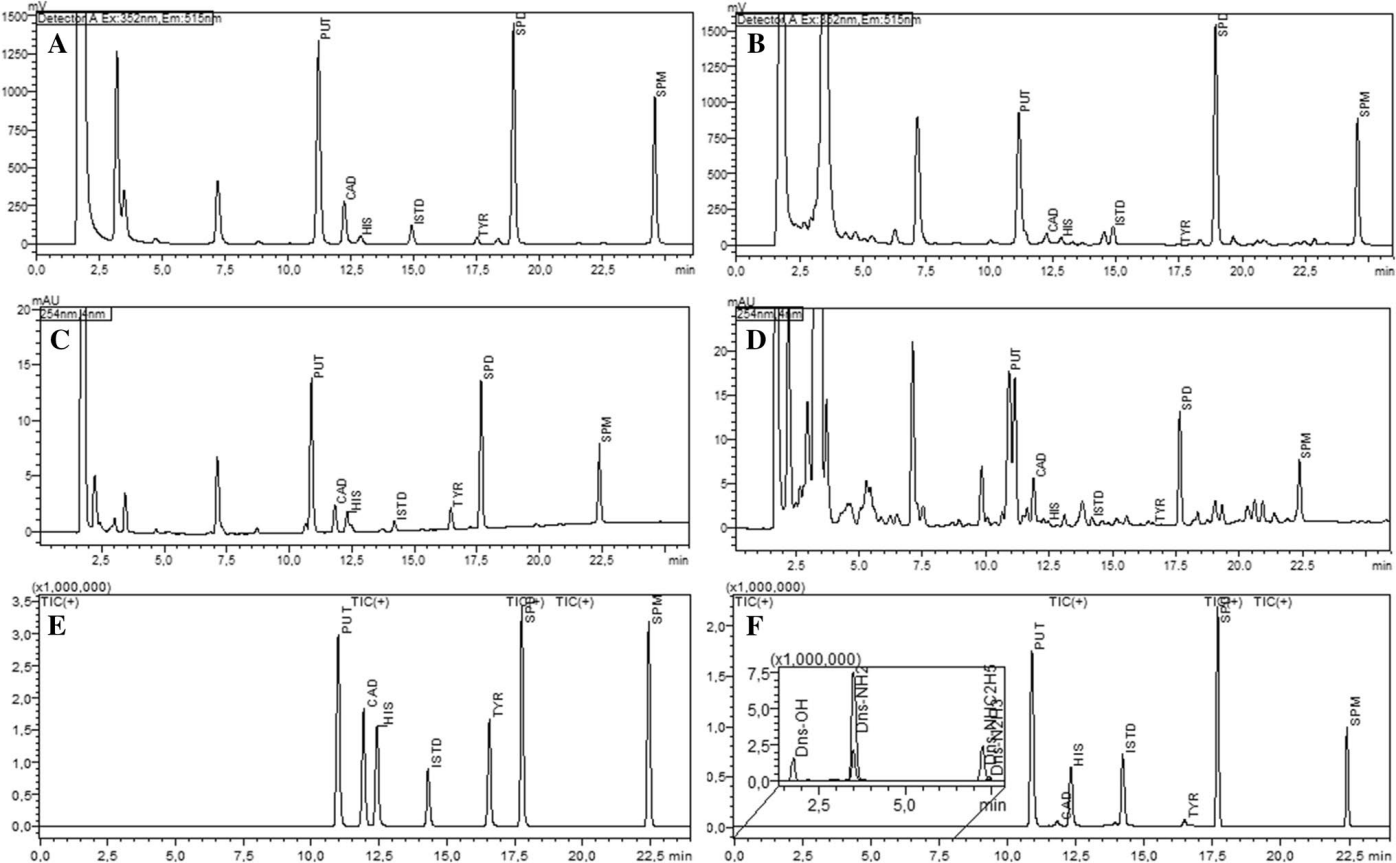
HPLC chromatograms of dansylated amines obtained for different detection systems: **a, b** FLD, **c, d** DAD and **e, f** APCI-MS SIM(+) operated in-line. Chromatograms **a, c, e** were obtained for a standard solution of BAs: PUT = 510, CAD = 97, HIS = 98, ISTD = 50, TYR = 99, SPD = 495, SPM = 303 ng mL^− 1^ and **b, d, f** for a vegetable sample with fish intended for infants

Determining the BAs content in food matrices containing low concentrations of HIS, TYR and CAD in comparison with PUT, SPD, and SPM, which occur in food products for children at much higher concentrations, presents certain difficulties, especially for classic detection systems such as UV or FL (Fig. 3a–d). First, side-products of the derivatization agent, dansyl chloride, show up on the chromatograms, and second, the significant differences in the concentrations of amines present in the samples impede their quantification. The side-products of Dns-amine reaction: dansyl acid (Dns-OH, [M+H]^+^ = 252), dansylamide (Dns-NH_2_, [M+H]^+^ = 251), dansyl hydrazine (Dns-N_2_H_3_, [M+H]^+^ = 266), *N*-dansyl ethylamine (Dns-NHC_2_H_5_, [M+H]^+^ = 279) (Fig. 3f) observed with UV and FL detection systems essentially elute before BAs, but some compounds present in the real samples also partially co-elute with PUT. The most promising results were obtained using the FL detection (Fig. 3a, b), but the low intensity of HIS and TYR fluorescence exclude its application with sufficient sensitivity for analysis of baby food. Other amine derivatization agents such as *o*-phthaldialdehyde/2-mercaptoethanol (only primary amines), or benzoyl chloride used in the pre-column mode with HPLC–DAD/FLD system (data not shown) could not be exploited for rewarding sensitivity level.

Using only a single-quadrupole MS detector in SIM mode, we could overcome both, the issue of differences in amine concentration in samples and the interferences caused by side-products of dansylation and co-eluting derivatives (Fig. 3e–f). Our method is fully adequate for the challenging task of determining BAs in baby foods, but requires careful optimization of the chromatographic system, ion source, and MS detector parameters to obtain desired sensitivity. To optimize the ionization of each BA in the source, various mobile phases were tested. In general, only acetonitrile was used as an organic phase modifier, in combination with different volatile salts with acid additives, to obtain the appropriate value of pH for ionization. All amines (gas-phase basicity > 200 kcal mol^− 1^) gave more intensive signals in mobile phase consisting of ACN with ammonium formate (gas-phase basicity 179 kcal mol^− 1^) than in ammonium acetate (gas-phase basicity 191 kcal mol^− 1^), and a higher signal was obtained with 10 mM salt concentration than with 15 or 20 mM. SPM, a polyamine similar to SPD but which elutes in most current MS methods [1, 10] as an irregular and low-intensity peak, had the highest intensity in ammonium formate mobile phase (pH ~ 5), due to its higher Δ energy. In this instance, no acid modifier (formic or acetic acid) was needed. The mobile phase gradient was optimized for the shortest analysis time with appropriate resolution of amines, which allowed for separation of all BAs in 24 min (30 min re-equilibration).

ESI is commonly used in analyses of BAs and amino acids for the ionization of highly polar compounds. To achieve proper ionization efficiency of the analyzed compounds, the ESI and APCI sources results were compared. The APCI source could be operated at higher mobile phase flow rates (up to 2 mL min^− 1^) and enabled a higher ionization temperature (up to 500 °C) to be used, resulting in minimum suppression of ionization and good ionization efficiency for BAs, without their needing to be pre-ionizable by acid additives in the mobile phase.

The APCI source parameters [nebulizing gas flow (NG) and drying gas flow (DG), temperature of APCI, heat block (HB) temperature, and desolvation line (DL) temperature] were optimized to achieve the best analytical conditions. The optimizations were performed by triple injection of the standard solutions of all amines, taking into account the changes in the composition of the mobile phase during the process in specified segments (Figs. S1–S5). The optimal source parameters were temperature for APCI = 450 °C (maximum setting value 500 °C was not used), for HB = 300 °C and for DL = 200 °C, and the optimal nitrogen flows for NG = 4.0 L min^− 1^ (maximum setting value 4.4 L min^− 1^ was not used due to the occurrence of a whistling sound) and for DG = 15 L min^− 1^. The APCI voltage was set at 4.5 kV, and the detector at 1.3 kV. The lowest ionization efficiency in the optimized method was obtained for SPM. Therefore, for this amine, the interface and detector voltages were increased to 5.0 and 1.5 kV, respectively. Optimization of the ion optic parameters for standard solutions of the amines using the LabSolution software was also performed. All experiments were conducted in triplicate. To quantify the BAs with appropriate sensitivity, the SIM(+) mode was used in four segments (Table 1).

**Table 1:** SIM(+) conditions for the APCI-MS method

Segment	Time (min)	Voltage (kV)		DL Volt, Qarray DC, Qarray RF (V)	[M+H]^+^ (*m*/*z*)
		APCI	Detector		
1	0.0–11.45	4.5	1.3	40, 30, 78	555.3
2	11.45–17.2	4.5	1.5	40, 30, 78	569.3, 578.3, 597.3, 604.2
3	17.2–19.0	4.5	1.3	40, 30, 104	845.3
4	19.0–24.0	5.0	1.5	40, 30, 130	1135.5

Using Dns-Cl as the pre-column derivatization agent resulted in the formation of stable derivatives with relatively high molecular masses, producing strong signal ions in positive mode. Furthermore, the high masses of the Dns-BAs precursor ions obtained enabled fragmentation with formation of the product ion, specific fragment of *m*/*z* 170 (5-(*N,N*-dimethylamino)naphthyl ion) [1].

In-source fragmentation of BAs to the *m*/*z* 170 ion in single-quadrupole MS was used to identify dansylated amines. In-source fragmentation was achieved by increasing the desolvation line (DL) and Qarray DC voltages (DL/DC) for segment 1: 40/70 V, segment 2: 60/90 V, segment 3–4: 60/100 V. In-source fragmentation was used to confirm identification of all dansylated amines in this study, yielding in fragment *m*/*z* 170 in a range from 1% for SPM, ISTD, 2% for TYR, CAD, 6–7% for SPD and HIS, to 10% for PUT of precursor ion intensity in mass spectra. Greater fragmentation of HIS occurred during in-source fragmentation than of other amines. This approach also confirmed the identification of peak which eluted after PUT in real samples (peak present in UV chromatograms (DAD) and in fluorescence detection (FLD) as a tailing peak) as dansyl derivative.

### Optimization of Sample Preparation Procedure

The application of a derivatization step, as well as the LLE clean-up procedure, was essential for quantification of BAs in almost all analyzed baby food samples.

**Fig. 4:**
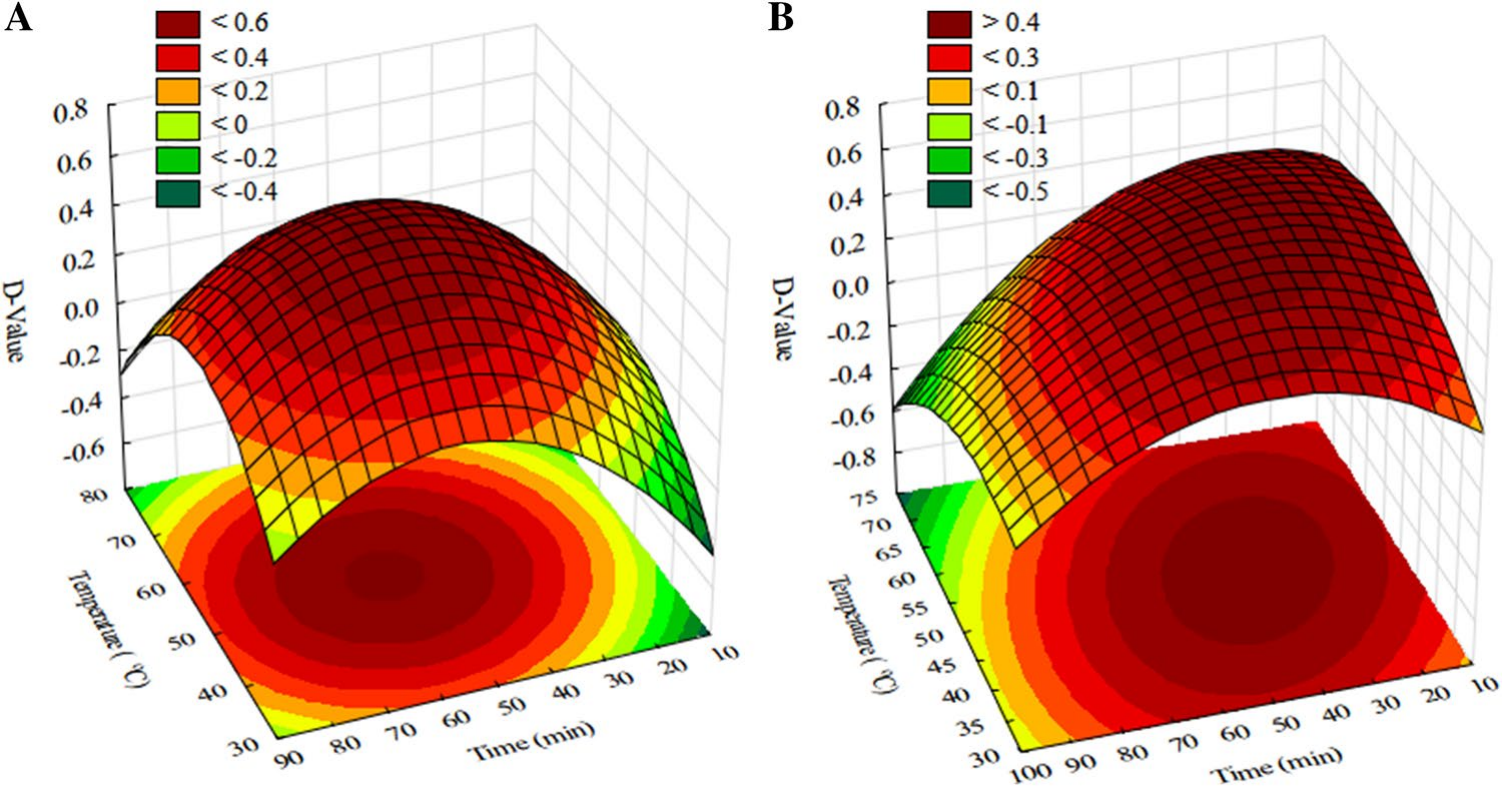
Desirable surface response as relative MS response for 7 BAs obtained for the optimization of dansylation time and temperature in **a** amine standard solution, **b** baby food sample (*n* = 3)

The optimization of the sample preparation procedure for baby foods included selection of the extraction solvent (5% TCA, 0.4 M HClO_4_ performed in 1 or 2 extraction steps—45 mL or 25 + 20 mL) (Fig. S6a), adjusting the pH (9.0, 10.0, 11.0, 11.5, and 12.0) (Fig. S6b) and modifying the temperature (40, 50, 60, and 70 °C) together with the reaction time (30, 45, 60, and 75 min) for higher dansylation efficiency (Fig. 4) and the conditions of LLE extraction (2 × 1 mL, 3 × 1 mL, 4 × 1 mL of diethyl ether) (Fig. S6c). The best conditions for BAs isolation from samples were using 0.4 M HClO_4_ as an acid extraction solvent added in a single volume of the process and the dansylation reaction, which was performed in pH 11.0 at 50 °C for 60 min. Most current methods involving the dansylation reaction are performed in pH = 9 [1, 20] or in generally alkaline medium [21]. The estimated results of pH optimization show that in pH = 11.0 amines such as HIS and CAD provide more intensive signals. Dns-Cl was dissolved in acetone because of difficulties related to the evaporation of acetonitrile frequently-used in other methods described in the literature. Certain results for these optimal dansylation parameters are in-line with those presented in the literature [1, 21]. The obtained derivatives were extracted with 3 × 1 mL of diethyl ether. With this method, there is no need to use a large volume of extraction solvent (only 3 mL per sample) and the emulsification which occurs during the LLE extraction step is overcome through centrifugation, resulting in high BA recovery values. This methodology enables the use of low sample dilution factor (the lowest equals 10), without preconcentration (LLE in a ratio of 1:1), because most matrix components, amino acids, and reagents remain in the aqueous phase.

### Method Validation Results

The calibration data, LODs, and LOQs are presented in Table 2. The sensitivity of the method is reflected by the LODs values ranged from 0.07 ng mL^− 1^ (HIS, CAD, and TYR) to 1.67 ng mL^− 1^ (SPD and SPM), which are lower than those reported in the literature, even for LC–MS/MS methods. The obtained 2.0 ng g^− 1^ LOQs for HIS, CAD, and TYR are out of range for any currently existing method of BAs analysis in food matrices. For soybean meal, LODs ranged from 14.9 ng g^− 1^ for TYR to 19.1 ng g^− 1^ for HIS using UHPLC–ESI–MS/MS [10]. For wines estimated, LODs ranged from 30.8 ng mL^− 1^ for CAD to 441 ng mL^− 1^ for TYR using HPLC–APCI–MS [22]. In donkey milk, estimated LODs ranged from 0.56 ng mL^− 1^ for TYR to 15.3 ng mL^− 1^ for SPM using HPLC–APCI–MS [21]. For fish, LODs ranged from 20 ng g^− 1^ for SPD to 250 ng g^− 1^ for SPM using HPLC–ESI–MS/MS [13].

**Table 2:** Analytical characteristics of the HPLC–APCI–MS method (*n* = 3)

Dansylamide	Retention time (min)	Linear range (ng mL^− 1^)	Calibration equation^a^	*R* ^2^	LOD^b^	LOQ^b^	LOQ^c^ (ng g^− 1^)	Amount added (ng mL^− 1^)	Intra-day, *n* = 3	Inter-day, *n* = 9	Recovery ± SD (%)
					(ng mL^− 1^)				(RSD, %)		
(Dns)_2_-PUT	11.01 ± 0.02	2.5–500	*y* = 0.351474*x −* 0.00249921	0.9996	0.80	2.5	25.0	25	2.2	2.5	100.6 ± 15.0
								127	1.7	3.9	102.5 ± 7.9
								255	1.8	2.7	103.6 ± 4.2
(Dns)_2_-CAD	11.95 ± 0.02	0.2–100	*y* = 1.13648*x* + 0.000709481	0.9999	0.07	0.2	2.0	5	3.6	4.1	100.0 ± 7.2
								24	2.9	4.1	98.8 ± 4.7
								48	3.4	3.1	100.1 ± 3.4
(Dns)_2_-HIS	12.45 ± 0.02	0.2–100	*y* = 0.977505*x* + 0.00704862	0.9999	0.07	0.2	2.0	5	4.9	3.2	102.1 ± 12.7
								25	6.3	9.7	90.6 ± 14.6
								49	7.3	8.0	91.2 ± 9.6
(Dns)_2_-TYR	16.59 ± 0.01	0.2–100	*y* = 0.908411*x* + 0.00548783	0.9998	0.07	0.2	2.0	5	1.8	1.4	97.9 ± 11.0
								25	6.5	7.5	91.4 ± 17.1
								50	3.8	5.3	95.7 ± 5.8
(Dns)_3_-SPD	17.79 ± 0.01	5.0–500	*y* = 0.346042*x* − 0.0195579	0.9995	1.67	5.0	50.0	25	2.6	2.7	105.2 ± 17.2
								124	7.5	4.9	97.0 ± 9.8
								248	4.4	3.9	95.7 ± 5.8
(Dns)_4_-SPM	22.48 ± 0.02	5.0–300	*y* = 0.528549*x* − 0.0219078	0.9990	1.67	5.0	50.0	24	1.8	3.8	100.8 ± 11.4
								121	2.6	5.1	86.4 ± 6.5
								243	3.9	5.8	86.0 ± 6.1

^a^Weighted least-squares linear regression (weighting factor *1*/*x*)

^b^For LC–MS technique

^c^For RP–HPLC–APCI–MS with Dns-Cl method in baby foods

For all the studied BAs, good linearity was obtained with *R*^2^ ranging from 0.9990 to 0.9999 (Table 2). Linearity was established over 2–3 orders of magnitude.

The relative standard deviation ranges of the components were 1.7–7.5% for intra-day analysis and 1.4–9.7% for inter-day analysis, indicating a good standard of precision (Table 2).

The developed method was reproducible with good recovery in the range of 86.0% for SPM and 105.2% for SPD with RSD ≤ 17.2% (Table 2).

Signal suppression as %SSE (signal suppression/enhancement) was observed for all BAs (100% SSE means that there is no matrix effect) (Table S1). Very low suppression, observed as a reduction in signal response, ranged from an estimated − 2 for CAD (98% SSE) to −  7 for SPM (93% SSE) was obtained. Mild signal suppression, over 10%, was estimated for TYR − 14 (86% SSE). Nevertheless, the SSE values obtained are relatively low and the estimated matrix-matched calibration curves did not differ significantly.

### Application of HPLC–APCI–MS Method to Baby Food Samples

**Fig. 5:**
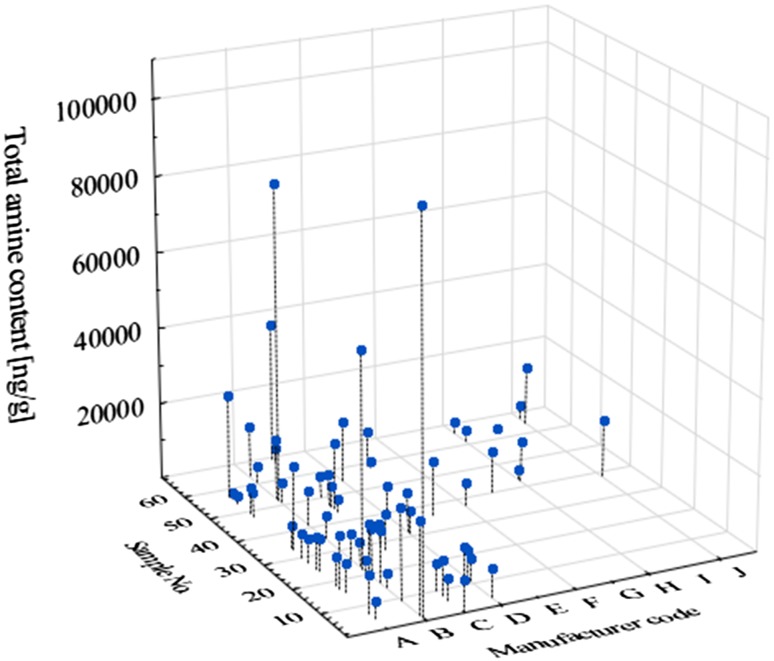
Profile of total amine content obtained for analyzed baby foods (*n* = 68)

The optimized method was applied to determine the content of 6 BAs in 68 samples of baby foods. HPLC–APCI–MS method was fully adequate for quantification of almost all selected amines present in the samples within a suitable concentration range (4/408 results were < LOQ). The summary amine levels in analyzed baby food products were found in a wide range of 1283–101423 ng g^− 1^ (Fig. 5). The particular amines were quantified as being in the range of 704–53416 ng g^− 1^ for PUT, 2–1263 ng g^− 1^ for CAD, 2–2375 ng g^− 1^ for HIS, 2–1668 ng g^− 1^ for TYR, 408–46680 ng g^− 1^ for SPD and 34–5619 ng g^− 1^ for SPM [23]. The application of highly-sensitive HPLC–APCI–MS method in amine analysis of baby foods allowed identification of food ingredients which may be necessary to remove (tuna, possibly spinach), reduce the amount added (spinach, green peas), either reduce its using by infants under 12 months of age (beef), or control the consumption (fruit baby products with banana) [23]. The obtained results enabled, for the first time, the assessment of a potential acute reference dose (ARfD), and the BAI (biogenic amine index) for baby foods [23].

## Conclusion

To the best of our knowledge, there has been no research to date to determine the biogenic amine profile of baby foods, due to extremely low limit of detection to be obtained. Using HPLC–APCI–MS and pre-column derivatization with dansyl chloride, we identified the BAs profile and quantified the most toxic ones (HIS, TYR) in commercial ready-to-eat baby foods at concentration levels as low as 2 ng g^− 1^, without requiring the use of SPE for clean-up and preconcentration with minimum use of organic solvents for the sample preparation. The optimized method is suitable for simultaneous analyses of PUT, CAD, HIS, TYR, SPD and SPM at low LODs. It provides significantly improved sensitivity when compared to MS and MS/MS methods described in the literature, particularly for CAD, HIS and TYR analysis, and enables in-source fragmentation to the product ion without using tandem mass spectrometry (use of low cost spectrometer). The presented method could be easily extended to other sample matrix with low amine levels, with the opportunity of high sample preconcentration during the LLE procedure, e.g., water or ice. The HPLC–APCI–MS method could be a milestone achievement, providing an essential analytical tool for the specific identification of components in baby foods with the most potential to provoke allergy-like responses and other adverse reactions.

## Electronic supplementary material

Below is the link to the electronic supplementary material.

Supplementary material 1 (DOC 165 KB)
