# Supplementary material for: Liquid Chromatography–Single-Quadrupole Mass Spectrometry as a Responsive Tool for Determination of Biogenic Amines in Ready-to-Eat Baby Foods
Source: Chromatographia. 2018 May 2;81(6):901–10. doi: 10.1007/s10337-018-3527-z (PMC5972156; doi:10.1007/s10337-018-3527-z)
Supplement: Supplementary file 1 — Supplementary material 1 (DOC 165 KB) [file 10337_2018_3527_MOESM1_ESM.doc]

**Liquid chromatography-single quadrupole mass spectrometry as a responsive tool for determination of biogenic amines in** **ready-to-eat** **baby foods**

**CHROMATOGRAPHIA**

**Anna Czajkowska-Mysłek*, Joanna Leszczyńska**

*corresponding author: Anna Czajkowska-Mysłek

Mass Spectrometry Laboratory, Wroclaw Research Centre EIT+, 147 Stabłowicka, 54-066 Wroclaw, Poland, Email: anna.czajkowskamyslek@gmail.com

Table S1

Evaluation of matrix effect by calculation of signal suppression/enhancement SSE (%) for BAs (n=3).

| Biogenic  amine | Slope | | R^2^ | | SSE  (%) |
| --- | --- | --- | --- | --- | --- |
|  | Solvent | Matrix-matched | Solvent | Matrix-matched |  |
| PUT | 0.351474 | 0.341565 | 0.9996 | 0.9966 | 97 |
| CAD | 1.13648 | 1.11275 | 0.9999 | 0.9989 | 98 |
| HIS | 0.977505 | 0.932824 | 0.9999 | 0.9955 | 95 |
| TYR | 0.908411 | 0.781095 | 0.9998 | 0.9938 | 86 |
| SPD | 0.346042 | 0.313698 | 0.9995 | 0.9930 | 90 |
| SPM | 0.528549 | 0.493074 | 0.9990 | 0.9805 | 93 |


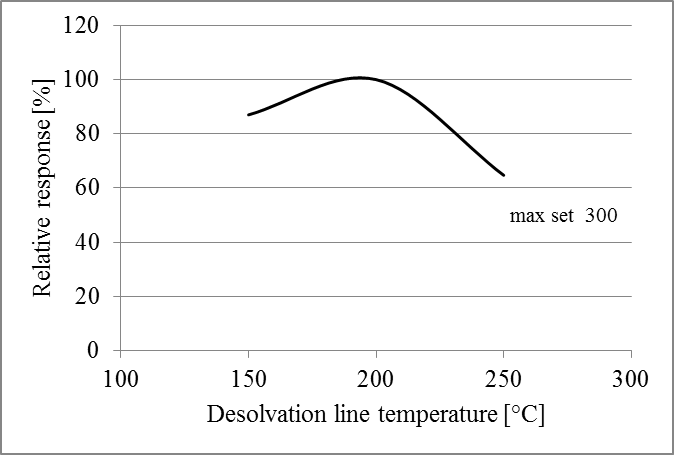


Figure S1. Relative MS response vs. desolvation line temperature.


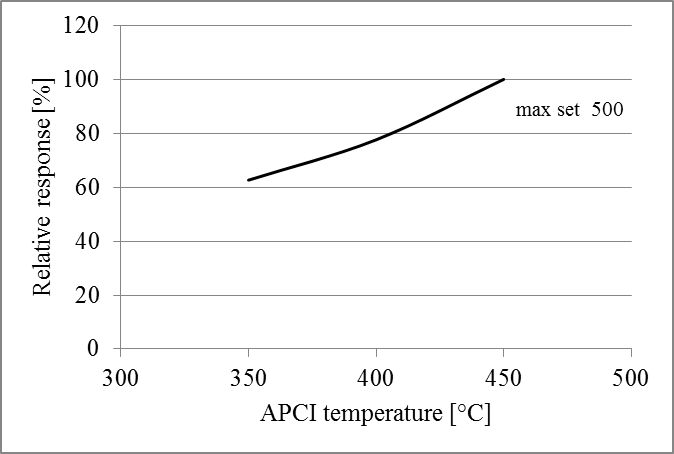


Figure S2. Relative MS response vs. APCI temperature.


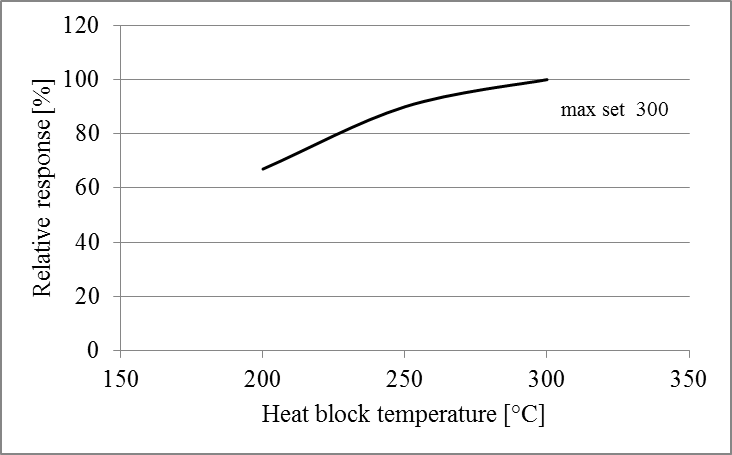


Figure S3. Relative MS response vs. heat block temperature.


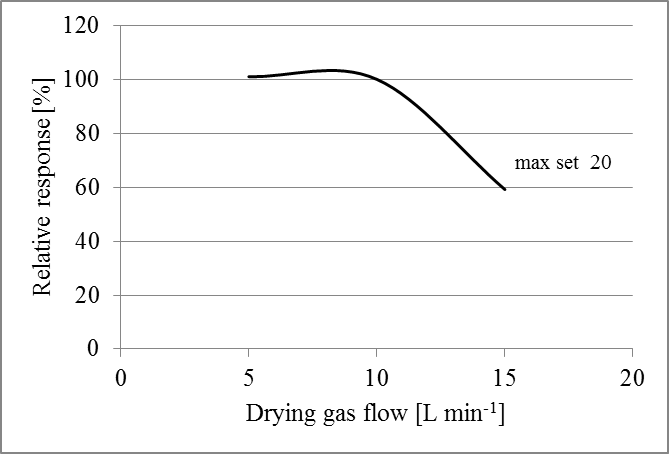


Figure S4. Relative MS response vs. drying gas flow (N_2_).


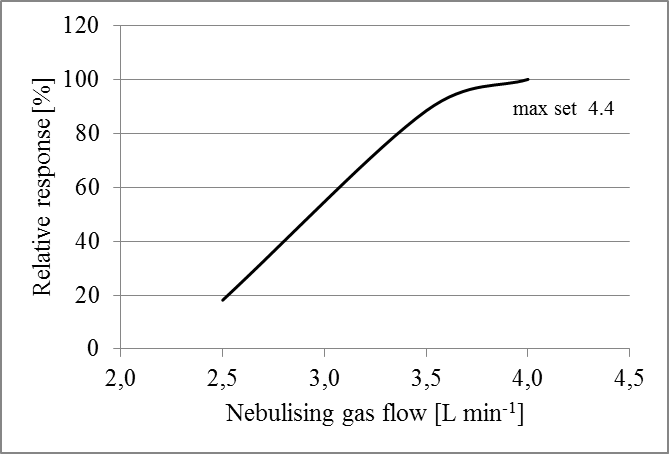


Figure S5. Relative MS response vs. nebulising gas flow (N_2_).


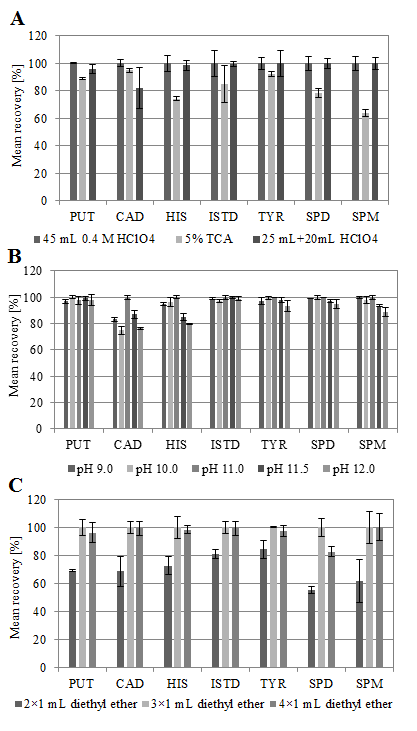


Figure S6. Mean recovery of 7 BAs from sample of baby food in relation to (A) different extraction conditions, (B) influence of derivatization pH (C) the number of diethyl ether LLE repetitions (n=3).
